# Coral bleaching due to cold stress on a central Red Sea reef flat

**DOI:** 10.1002/ece3.9450

**Published:** 2022-10-22

**Authors:** Walter A. Rich, Susana Carvalho, Michael L. Berumen

**Affiliations:** ^1^ Red Sea Research Center King Abdullah University of Science and Technology Thuwal Saudi Arabia

**Keywords:** aerial exposure, cold stress, *Stylophora pistillata*

## Abstract

Ocean warming is leading to more frequent coral bleaching events. However, cold stress can also induce bleaching in corals. Here, we report observations of a boreal winter bleaching event in January 2020 in the central Red Sea, mainly within a population of the branching coral *Stylophora pistillata* on an offshore reef flat. Sea surface temperatures (SSTs) rarely fall below 24°C in this region, but data loggers deployed on several nearby reef flats recorded overnight seawater temperatures as low as 18°C just 3 days before the observations. The low temperatures coincided with an extremely low tide and cool air temperatures, likely resulting in the aerial exposure of the corals during the night time low‐tide event. The risk of aerial exposure is rare in winter months, as the Red Sea exhibits seasonal fluctuations in sea level with winter values typically 0.3–0.4 m higher than in summer. These observations are notable for a region typically characterized as a high‐temperature sea, and highlight the need for long‐term monitoring programs as this rare event may have gone unnoticed.

## Graphical abstract

Coral bleaching is most often caused by high temperatures, but it can also be caused by cold stress. Here, we report observations of coral bleaching on a shallow reef flat in the central Red Sea coinciding with unusually low temperatures. These observations are notable for a region typically characterized as a high‐temperature sea.

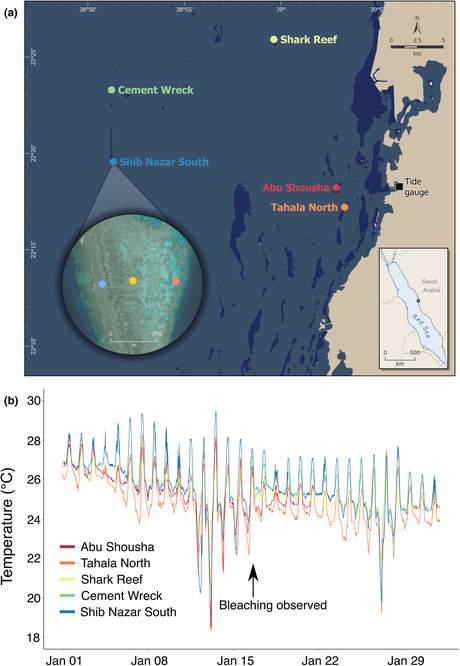

## 1 INTRODUCTION

Coral bleaching events are becoming more frequent in recent years (Hughes et al., 2018). Bleaching is a stress response that results in corals expelling their algal symbionts due to a stressful event. Although local stressors such as excess nutrients, pollution, or sedimentation can induce bleaching, the principal cause is high temperatures (Suggett & Smith, 2020). As the oceans continue to warm due to anthropogenic impacts, many coral reefs are predicted to bleach annually by the end of this century (Van Hooidonk et al., 2016).

While bleaching due to warm conditions is well documented, cold temperature stress can also cause corals to bleach (Bellworthy & Fine, 2021; Hoegh‐Guldberg et al., 2005; Hoegh‐Guldberg & Fine, 2004; Kemp et al., 2011; Marangoni et al., 2021; Paz‐García et al., 2012; Pontasch et al., 2017; Saxby et al., 2003; Tuckett & Wernberg, 2018). The mechanisms causing the bleaching response to both heat and cold stress appear to be similar, and are related to reactive oxygen species forming in coral tissues (Marangoni et al., 2021). Typically, cold stress is limited to high latitude or marginal coral reef systems (Tuckett & Wernberg, 2018). Bleaching due to cold conditions is much less frequently recorded than warm‐water bleaching events, but it can have major impacts on coral communities. For example, a cold spell in the Florida Keys caused nearly 100% mortality of several coral species on nearshore reefs (Kemp et al., 2011). Although bleaching due to heat stress is more likely in a warming world, cold stress may become more frequent in the future as the climate becomes more unstable and results in extremely cold weather events (Johnson et al., 2018).

The Red Sea is one of the warmest seas in the world (Berumen et al., 2019). In the central Red Sea, remotely sensed SSTs rarely fall below 24°C even in winter months (Shaltout, 2019). Coral reef organisms in the central Red Sea typically experience a moderate annual temperature range, with a year‐long monitoring study showing in situ temperatures ranging from 25 to 32°C for an offshore reef (Roik et al., 2016). However, in specific or marginal habitats, such as the reef flat, temperature ranges are more extreme and can occasionally exceed 37°C for brief periods in the afternoon during summer months (Davis et al., 2011; Rich et al., 2022). Recent studies from the central Red Sea have highlighted that local corals are capable of tolerating these high temperatures for short periods of time (Anton et al., 2020; Evensen et al., 2022; Voolstra et al., 2020, 2021). Despite this, the region has experienced several coral bleaching events due to heat stress in recent years (Furby et al., 2013; Monroe et al., 2018). However, owing to its comparatively warm winter temperatures, bleaching due to cold stress is rare in the central Red Sea. Here, we report observations of a bleaching event during January 2020 on the reef flat of an offshore reef in the central Red Sea. The observations followed a period of unusually cool weather coupled with an extremely low tide, which we hypothesize caused the bleaching event on the shallow reef flat.

## 2 MATERIALS AND METHODS

### 2.1 Study site

**FIGURE 1:**
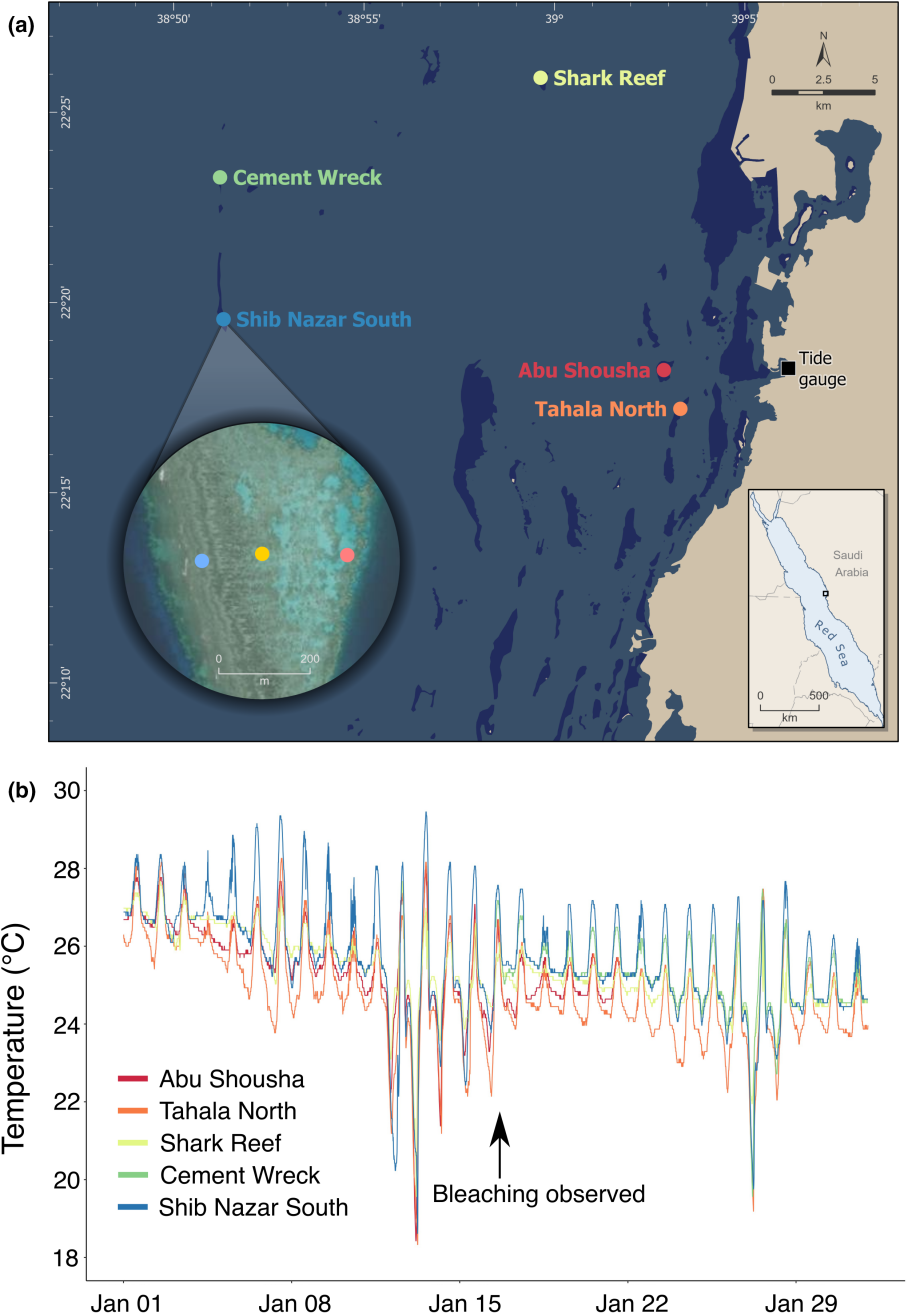
(a) Map of the study area. Bleaching was observed on Cement Wreck but it did not have temperature loggers deployed at the time. We used temperature data from nearby reefs to infer the possible conditions experienced by corals on Cement Wreck before the observations were made. The inset is of Shib Nazar south reef flat where temperature loggers were deployed on the exposed, midreef and sheltered zones (blue, yellow, and red circles, respectively). (b) Temperature profiles from five reefs in the study area during January 2020. All the temperature profiles are from the midreef logger site of each reef (e.g., the yellow point in the center of the reef flat shown in the inset of Shib Nazar south).

The observations were made on the reef flat of Cement Wreck, which is situated approximately 20 km offshore in the central Saudi Arabian Red Sea (Figure 1a). Cement Wreck is a site that is part of the Shib Nazar reef system, which is roughly 10 km in length and is oriented approximately in a north–south direction. The reef flat lies at a depth typically of 0.5–1.5 m, though extreme events can cause even shallower depths. The Red Sea generally experiences low sea level variability, but it varies seasonally with average depths tending to be greater in the winter (Abdulla & Al‐Subhi, 2021; Churchill et al., 2019). The reef flats in the region are dominated by *Stylophora pistillata* (Rich et al., 2022) and it appeared to be the sole branching species on the Cement Wreck reef flat.

### 2.2 Data collection

**FIGURE 2:**
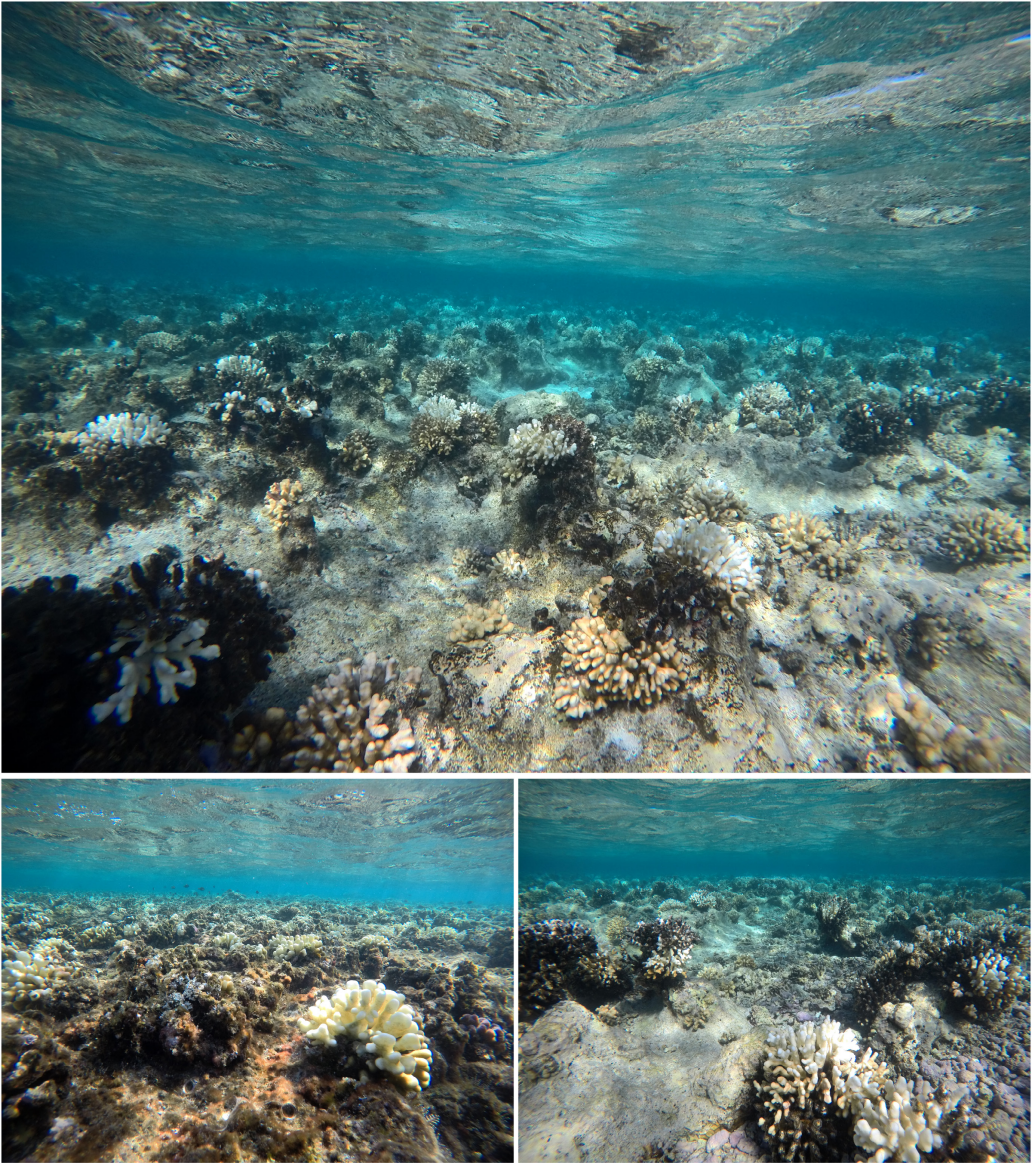
In situ photographs of the coral bleaching on the reef flat of cement wreck. The photos were taken on January 16, 2020. Bleaching, mainly of *Stylophora pistillata*, was widespread throughout the entire reef flat.

As a part of an ongoing monitoring program, temperature loggers (Onset, Bourne, US) were deployed in June 2019 across several reef flats over a cross‐shelf gradient (Figure 1a). The loggers were placed on the shallow reef flat and recorded temperature every 10 minutes. On January 16, 2020, we visited the reef flat of Cement Wreck to deploy loggers as part of this program; until then, no in situ temperature data were available for this site. We noted coral bleaching on the reef flat and thus made bleaching observations opportunistically. We took several photos with a GoPro Hero 5 (GoPro Inc.) to document the event (Figure 2) but did not make any quantitative measurements of bleaching. In addition to deploying temperature loggers, we deployed an acoustic doppler current profiler (ADCP) (Nortek, Rud, Norway) to record current data at a frequency of 30 minutes. The ADCP also records pressure and thus tidal fluctuations can be calculated from each measurement. We also acquired tidal data from a tide gauge (Valeport 740, Totnes, UK) deployed in the harbor of the King Abdullah University of Science and Technology (KAUST), roughly 27 km east of the Cement Wreck. The tide gauge recorded data at a frequency of 10 min.

Although we do not have in situ temperature data for Cement Wreck prior to January 16, 2020, we can compare the in situ data from nearby reefs to gain insight into the possible conditions experienced by corals on Cement Wreck prior to our observations. Shib Nazar South is another site that is part of the same reef system and is situated about 7 km due south of the Cement Wreck site. Shib Nazar South had temperature loggers deployed on its exposed, midreef and sheltered reef flat zones prior to the bleaching event (Figure 1a inset). In this region, wave‐driven water flow generally moves from west to east over the reef flat, and heats up as it moves towards the protected side of the reef (Davis et al., 2011). We plotted the temperature readings of all three Shib Nazar South loggers for January 2020 to visualize the seawater temperature at different locations over the reef flat. We also compared the midreef loggers of four other reefs with Cement Wreck for the rest of January 2020 to assess if they follow similar temperature patterns, and whether temperatures on Shib Nazar South (the nearest site) are a good proxy for what occurred on Cement Wreck before the bleaching observations were made. In addition, we wanted to assess if the minimum temperatures from January 2020 were typical in winter for Cement Wreck and other reefs in the wider complex of reefs in the region. We extracted minimum daily temperatures from the midreef loggers of all reefs from June 2019 to April 2022.

In addition to the in situ temperature data, we extracted satellite data to investigate the wider oceanographic and atmospheric conditions prior to the bleaching observations. We extracted hourly data for SST and 2 m air temperature for January 2020 from the European Center for Medium‐Range Weather Forecasts ERA5 reanalysis data. We used the previous 30 years of data (1989–2019) to calculate an hourly climatology of both SST and 2 m air temperature for the month of January.

## 3 RESULTS AND DISCUSSION

Coral bleaching due to cold stress has been documented in several regions (Hoegh‐Guldberg et al., 2005; Hoegh‐Guldberg & Fine, 2004; Kemp et al., 2011; Paz‐García et al., 2012). However, bleaching due to cold stress is rare in the central Red Sea. Although mortality of shallow water corals due to aerial exposure has been documented for the northern Red Sea, it occurred in the warmer periods and during daylight when intense irradiance could have caused heat stress and desiccation (Fishelson, 1973; Loya, 1975). In contrast, the extreme low‐tide event documented here occurred at night in conjunction with cool temperatures.

Although there are differences in the temperature ranges of the five reefs in the study area, all reefs broadly experience the same patterns (Figure 1b). The four reefs that had loggers deployed on January 13, 2020 all experienced similar minimum temperatures (Abu Shousha: 18.43°C; Tahala North: 18.33°C; Shark Reef 19.56°C; Shib Nazar South: 18.62°C). Cement Wreck did not have temperature loggers deployed during the minimum temperature event, but its temperature profile tracks closely with the other reefs from January 16, 2020 onward. In particular, all the reefs experienced similar temperatures during another low‐temperature event on January 27, 2020 (Cement Wreck: 19.56°C; Tahala North: 19.18°C; Shark Reef: 21.95°C; Shib Nazar South: 19.76°C). The temperature profile of Cement Wreck is very similar to the temperature profile from its nearest site, Shib Nazar South. Therefore, we used the temperatures measured at Shib Nazar South as a proxy for the conditions that occurred on Cement Wreck in the days prior to the bleaching observations.

**FIGURE 3:**
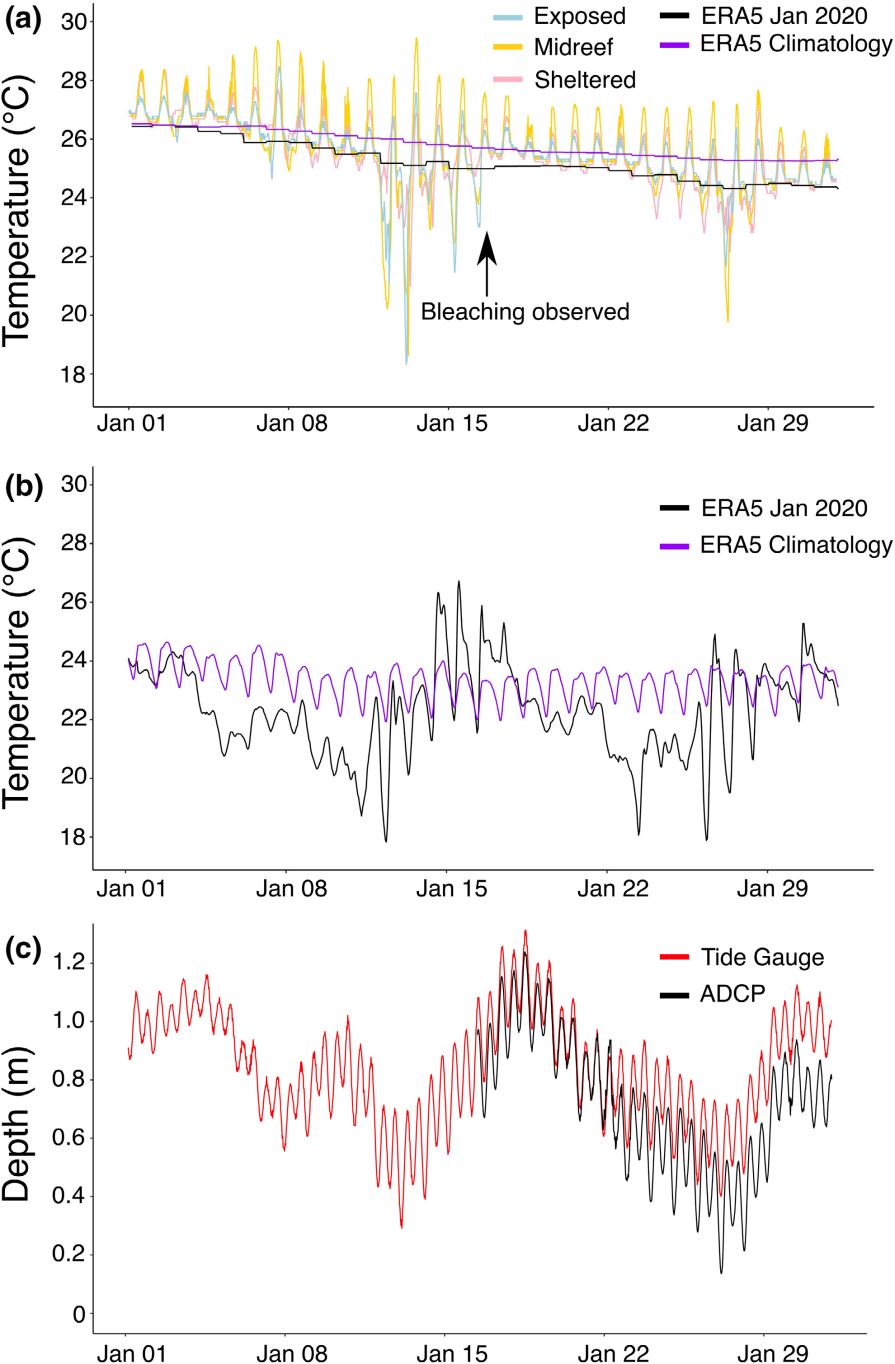
In situ and satellite‐derived data for January 2020 in the study area of Cement Wreck. (a) In situ temperature readings of a nearby site, Shib Nazar south, at three locations over the reef flat and ERA5 satellite‐derived SST for the grid cell encompassing the study area (January 2020 compared to 30‐year climatology). (b) ERA5 2 m air temperatures for the same grid cell (January 2020 compared to 30‐year climatology). (c) Water depth measured by an ADCP deployed on the Cement Wreck reef flat compared to measurements of a tide gauge deployed in the KAUST harbor.

The minimum temperatures recorded on the nearby reef flat of Shib Nazar South were 18.33°, 18.62°, and 21.00°C for the exposed, midreef and sheltered reef flat zones, respectively (Figure 3a). These all occurred in the early morning hours of January 13, 2020, but not at exactly the same time (03:30 a.m., 05:50 a.m., and 07:50 a.m. for exposed, midreef, and sheltered reef flat zones, respectively). The ERA5 satellite data for the grid cell encompassing Cement Wreck and Shib Nazar South shows the SST never declined below 24°C during the entire month of January 2020 (Figure 3a). This illustrates that the satellite‐derived data cannot capture the fine‐scale temperature fluctuations over shallow reef flats, nor does it detect diurnal changes in temperature. However, the ERA5 2 m air temperature data does detect hourly changes in air temperature over the same area (Figure 3b). The low temperatures recorded on the reef flat coincide with spring tides on January 13 and 27, 2020. Both events are captured by the KAUST tide gauge, with the later event also being recorded by the ADCP on Cement Wreck (Figure 3c).

The dominant coral inhabiting reef flats in the region is *S. pistillata*, with colony density averaging 6.33 individuals m^−2^ on Cement Wreck (Rich et al., 2022). This species appeared to be the main coral affected, with bleached colonies found across the reef flat (Figure 2). Previous studies of *S. pistillata* in the Red Sea have pointed to a minimum critical temperature around 18°C (Bellworthy & Fine, 2021), which agrees with our observations. In particular, symbiont physiology is affected in cold conditions (reduced photosynthetic efficiency, symbiont density, and chlorophyll content) (Bellworthy & Fine, 2021; Marangoni et al., 2021; Pontasch et al., 2017). Bleaching of *S. pistillata* because of cold stress appears to be an acclimation response to oxidative stress that may be induced by the symbionts (Marangoni et al., 2021), and can occur after only a few hours of exposure (Hoegh‐Guldberg & Fine, 2004). In a study of *S. pistillata* under cold stress conditions for seven days, there was no appreciable effect on host coral physiology (Bellworthy & Fine, 2021), but an experiment for 30 days noted reduced host protein content (Marangoni et al., 2021). The bleached colonies observed here likely harbored symbionts of the genus *Symbiodinium* (formerly clade A) (Hume et al., 2020), which are prevalent in shallow‐water colonies of *S. pistillata* in the central and northern Red Sea (Hume et al., 2020; Lampert‐Karako et al., 2008; Voolstra et al., 2020; Winters et al., 2009).

There is also the possibility that corals on this reef flat experienced aerial exposure due to a low‐tide event in tandem with the cold stress event. The tide gauge in the KAUST harbor recorded a low‐spring tide (0.29 m) that coincided with the low temperature reading in the early morning of January 13, 2020 (Figure 3c). Exposure to air can have negative effects on corals (Fishelson, 1973; Loya, 1975), which can be particularly impactful when coupled with low temperatures (Hoegh‐Guldberg et al., 2005; Hoegh‐Guldberg & Fine, 2004). In fact, aerial exposure during a winter cold spell in the Arabian Gulf caused total mortality of *Stylophora* on nearshore patch reefs (Fadlallah et al., 1995). The next spring tide at Cement Wreck occurred on January 27, 2020, but the minimum depth recorded by the tide gauge was 0.1 m higher than the event on January 13, 2020. The ADCP was not yet deployed on January 13, 2020, but on January 27, 2020 it recorded a minimum water depth of 0.14 m over the Cement Wreck reef flat. The pressure readings from the ADCP and tide gauge data follow similar patterns, and we presume the reef flat of Cement Wreck may have experienced similar, if not lower, water levels on January 13, 2020 before the ADCP was deployed. Such low sea level likely resulted in aerial exposure of at least the upper portions of the coral colonies, perhaps during both of the January spring tides. Aerial exposure due to extreme low tides has occasionally been reported in the northern Red Sea (Fishelson, 1973; Kotb et al., 2004; Loya, 1975), but it rarely occurs in winter months as sea level tends to be 0.3–0.4 m higher due to seasonal changes in wind patterns (Abdulla & Al‐Subhi, 2021; Churchill et al., 2019).

**FIGURE 4:**
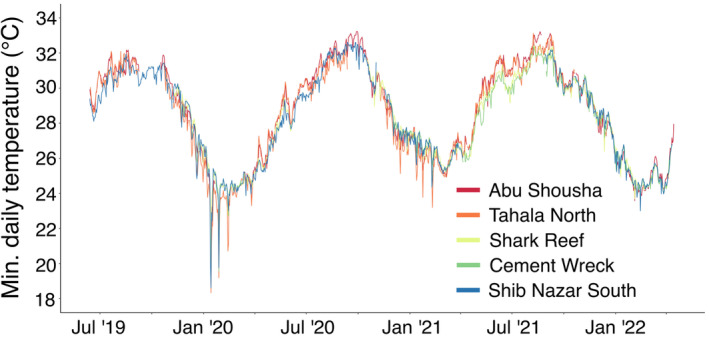
Minimum daily temperatures of five reef flats in the study region from June 12, 2019 to April 14, 2022. Data are from temperature loggers deployed in the midreef zone of each reef flat.

It is not known how frequently winter bleaching events may occur in the central Red Sea. In this region, long‐term monitoring programs are non‐existent, and we only have continuous in situ data for the region from June 2019 until April 2022. Plotting the daily minimum temperatures for that period, it is apparent that the winter of 2019–2020 experienced much cooler temperatures compared to the following two winters (Figure 4). Both the ERA5 SST and 2 m air temperatures for January 2020 were cooler than the 30‐year average for the respective parameters (Figure 3a,b). This combination of cool air temperatures and low‐sea level seems to be a rare occurrence, and previous bleaching events might have been unnoticed.

The bleaching event appeared to only affect corals inhabiting the shallow reef flat, as we did not observe any bleaching in deeper areas along the reef slope. However, it is unclear what further impacts the bleaching event had on the population of *S. pistillata*. We revisited Cement Wreck on Feburary 6, 2020 and the colonies appeared to be recovering (no white or recently dead colonies were observed; most colonies were pale yellow). Potential sublethal effects may have occurred (Baird & Marshall, 2002), but were not measured in this study. *Stylophora pistillata* from the Red Sea has been shown to be resilient to temperature extremes, but its tolerance to temperature stress is often diminished when co‐occurring with other stressors (Meziere et al., 2022).

The observations of bleaching due to cold stress reported here are notable for a region typically characterized as a high‐temperature sea, and highlight the need for long‐term monitoring programs. Given the apparent quick recovery of *S. pistillata* colonies, it is important to continuously monitor reefs in the region, as we may have missed this event entirely if we did not visit the site in January 2020. Although the population seemed to recover, it is unknown what possible sublethal effects this bleaching event may have had (e.g., reduced energy reserves or reproductive output) (Baird & Marshall, 2002), which may influence the response to other subsequent stress events. In addition, we were not able to visit any other reef flats in the period between January 13 to Feburary 6, 2020, and therefore, we do not know how widespread bleaching was on other reef flats. However, the other reef flats have similar coral communities dominated by *S. pistillata* and are of similar depth, so it is likely bleaching was more widespread than what was noted here. These observations also underscore the extreme conditions faced by corals on reef flats of the Red Sea, which must tolerate a wide temperature range and occasional aerial exposure. Considering the dire predictions coral reefs face under future climate change, future studies should explore the mechanisms by which these shallow‐water corals persist under extreme conditions.

## AUTHOR CONTRIBUTIONS

**Walter A. Rich:** Conceptualization (lead); data curation (lead); formal analysis (lead); investigation (lead); methodology (lead); visualization (lead); writing – original draft (lead); writing – review and editing (lead). **Susana Carvalho:** Formal analysis (supporting); methodology (supporting); supervision (equal); writing – review and editing (supporting). **Michael L. Berumen:** Funding acquisition (lead); supervision (equal); writing – review and editing (supporting).

## Data Availability

Data is available from the Dryad data repository: https://doi.org/10.5061/dryad.00000006g.
